# Apolipoprotein A1-Related Proteins and Reverse Cholesterol Transport in Antiatherosclerosis Therapy: Recent Progress and Future Perspectives

**DOI:** 10.1155/2022/4610834

**Published:** 2022-01-10

**Authors:** Xiuting Xu, Zikai Song, Bao Mao, Guoliang Xu

**Affiliations:** Department of Cardiology, The First Hospital of Jilin University, Changchun, Jilin 130000, China

## Abstract

Hyperlipidemia characterized by abnormal deposition of cholesterol in arteries can cause atherosclerosis and coronary artery occlusion, leading to atherosclerotic coronary heart disease. The body prevents atherosclerosis by reverse cholesterol transport to mobilize and excrete cholesterol and other lipids. Apolipoprotein A1, the major component of high-density lipoprotein, plays a key role in reverse cholesterol transport. Here, we reviewed the role of apolipoprotein A1-targeting molecules in antiatherosclerosis therapy, in particular ATP-binding cassette transporter A1, lecithin-cholesterol acyltransferase, and scavenger receptor class B type 1.

## 1. Introduction

Coronary artery disease (CAD) is a major public health concern because of the associated high morbidity, mortality, and disability, which increases the economic and psychological burden of patients. Atherosclerosis (AS), the main pathological basis for the development of CAD, refers to systemic lesions with age-related changes caused by the formation of fibrous plaques blocking the lumen, resulting in local tissue ischemia [1]. Therefore, the prevention and treatment of AS are major public health priorities [2].

Numerous studies have confirmed that abnormal lipid metabolism is a major risk factor and pathological basis for AS formation. In the early stages of atherogenesis, plasma cholesterol is abnormally deposited and high levels of lipoproteins and low-density lipoprotein (LDL) cholesterol-containing apoB in the blood cause damage to endocrine cells; LDL carries a large amount of cholesterol and undergoes oxidization to form oxidized LDL (Ox-LDL) in the vascular endocrine gap, further promoting lipid deposition [3]. At the same time, scavengers on the surface of macrophages can quickly identify and engulf Ox-LDL, evolving into foam cells containing large amounts of cholesterol esters (CEs), which accumulate in large quantities to form atherosclerotic lipid plaques [3, 4]. Foam cells gradually disintegrate to form an unstable, lipid-rich core with a rupture-prone fibrous cap. Unstable plaques develop cracks, erode, or rupture, resulting in thrombosis and, subsequently, acute target organ damage [5]. The reverse cholesterol transport (RCT) pathway is the major mechanism inhibiting AS formation via the transport of abnormally deposited cholesterol from peripheral tissue cells, including foam cells in atherosclerotic plaques, to the liver for excretion [6].

**Figure 1:**
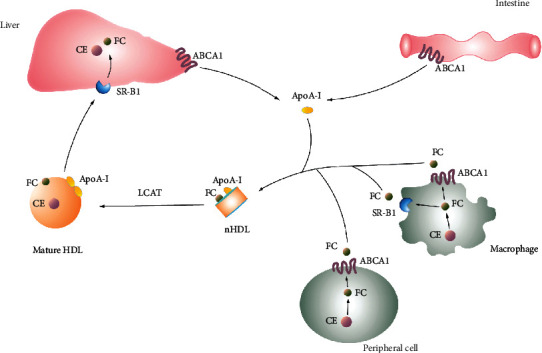
Diagram of reverse cholesterol transport.

High-density lipoproteins (HDLs) transport insoluble lipids and are the only plasma lipoproteins with anti-AS effects. They are synthesized mainly in the liver and intestines, are the smallest and densest type of plasma lipoprotein (diameter = 7–12 nm; density = 1.063–1.250 g/mL), and are composed of phospholipids, free cholesterol, CEs, and apolipoprotein A1 (apoA1), which is the main structural protein, accounting for approximately 70% of the protein content of HDL [7]. According to the size and mass-to-charge ratio of apoA1, the various types of particles containing apoA1 can be classified into pre-*β*-1 (including fat-free and fat-poor apoA1), pre-*β*-2, *α*-4, *α*-3, *α*-2, *α*-1, and pre-*α* substances [8]. HDL not only has anti-inflammatory, antioxidant, antithrombotic, and antiapoptotic effects but also mainly removes excess cholesterol via the RCT pathway, thus inhibiting the progression of AS [9–11]. apoA1, the main component of HDL, interacts with different receptors and transporters, including ATP-binding cassette transporter A1 (ABCA1), lecithin-cholesterol acyltransferase (LCAT), and scavenger receptor class B type 1 (SR-B1), and plays an important role in eliminating excess cholesterol. ABCA1 mediates the transfer of cholesterol to either fat-free or fat-poor apoA1, resulting in the production of cholesterol-rich new HDL (nHDL) [12], which is then esterified into mature, CE-rich spherical HDL under the action of LCAT [13] (Figure 1). Finally, lipids carried by HDLs (including free cholesterol and CE) are selectively removed by the hepatic HDL receptor, SR-B1 [14] (Figure 1). Various studies have found that the apoA1-binding receptors, ABCA1, LCAT, and SR-B1, may exert anti-AS effects through the modulation of inflammation and oxidative stress. These receptors may become major targets of anti-AS therapy in the future. Therefore, this paper will provide a detailed review of the research progress on the role and mechanism of ABCA1, LCAT, and SR-B1 in anti-AS therapy.

## 2. ABCA1

ABCA1 is a complete membrane protein composed of 2261 amino acids. The coding gene, which spans 149 kb, is located on 9q22-q31 and contains 50 exons and 49 intron groups. It is expressed mainly in hepatocytes, intestinal epithelial cells, and macrophages and contains two transmembrane domains (TMDs) [15]. Each TMD consists of six transmembrane (TM) helices, followed by a cytoplasmic region containing a nucleotide-binding domain (NBD) and a small regulatory domain [16]. In addition, ABCA1 has two characteristic extracellular domains (ECDs), namely, ECD1 between TM1 and TM2 and ECD2 between TM7 and TM8 [16]. Two intramolecular disulfide bonds are formed between these domains, which are necessary for apoA1 binding and HDL formation [17, 18]. ABCA1 is a key membrane-associated lipid transporter that maintains intracellular lipid homeostasis, plays an important role in preventing the accumulation of excess cholesterol in the cell membrane, and has an antiatherosclerotic effect.

The formation of nHDLs mediated by ABCA1 and apoA1 is the first and rate-limiting step in the RCT pathway, which plays an important role in initiating the development of early atherosclerotic lesion [12, 19]. Ishigami et al. believe that the main pathway for the formation of nHDL involves the transfer of phosphatidylcholine and cholesterol by ABCA1 monomers to the ECD in an ATP-dependent manner; when the ECD accumulates enough phosphatidylcholine and cholesterol, conformational changes occur, such as the formation of dimers, which prevents their diffusion; lipid-free apoA1 molecules can bind directly to the ECD of ABCA1 dimer and are loaded with lipids informing the ECD to form nHDL; and finally, the ABCA1 dimer releases chelated lipids and decomposes into monomers [18].

### 2.1. Anti-AS Effects of ABCA1

There is a negative correlation between the occurrence of CAD and the plasma levels of HDL cholesterol (HDL-C), and ABCA1 is one of the most important lipid transporters in the RCT affecting plasma HDL-C levels. A 50% increase in ABCA1-mediated cholesterol efflux can lead to a 30% increase in HDL-C concentration, thereby reducing the risk of CAD by 35% to 50%; thus, the study of ABCA1 has revealed potential novel approaches to the prevention and treatment of AS [20].

Studies have shown that ABCA1 dysfunction can lead to Tangier disease, characterized by severe HDL deficiency, lipid deposition in tissue macrophages, and generalized AS [21]. Furthermore, a lack or reduced activity of ABCA1 can lead to accelerated development of AS [22, 23]; carriers of the dysfunctional ABCA1 mutation have an increased risk of developing AS [24]. Overexpression of ABCA1 in mice and endothelial cells increases HDL-C and apoA1 levels in plasma and promotes RCT to the liver and biliary cholesterol excretion, which significantly reduces lipid deposition and halts the progression of AS [25, 26]. Overall, these studies indicate that ABCA1 plays an important structural and functional role in lipid metabolism and is a potential novel target of anti-AS therapy.

### 2.2. Recent Progress in the Anti-AS Applications of ABCA1

Rutaecarpine [27], pyrrole-imidazole polyamides [28], kinesin-binding protein 2 (TRAK2) [29], CXC chemokine ligand 12 (CXCL12) [30], and myocardin (MYOCD) [31] are novel gene regulators that prevent AS by regulating the ABCA1 gene promoter, which provides a new strategy for the treatment of atherosclerotic cardiovascular diseases. The key regulators of lipid metabolism-related gene expression, including microRNA (miRNA) and long noncoding RNA (lncRNA), regulate ABCA1 and plasma HDL-C levels after transcription [32, 33]. The expression of ABCA1 is targeted by many miRNAs in different cell types [34]; the downregulation of microRNA-17-5p (miR-17-5p) inhibits lipid accumulation and upregulates ABCA1, and it has been found that ABCA1 becomes the target of miR-17-5p by binding directly to the 3-untranslated region (3′-UTR) of miRNA [35]. Moreover, targeting microRNA-144 (miR-144) with antisense oligodeoxynucleotides may enhance RCT and cholesterol metabolism in patients with cardiovascular disease [36].

A recent study proposed a new mouse model that produces a specific point mutation at the microRNA-33- (miR-33-) binding site in the 3′-UTR of ABCA1, which blocks the binding of miR-33, resulting in increased expression of ABCA1 and cholesterol efflux and a decrease in foam cell formation [37]. The results suggest that inhibition of ABCA1 may be the main reason for miR-33 to accelerate atherogenesis and demonstrated for the first time that the destruction of a single miRNA/target interaction is sufficient to simulate the effects of miRNA deletion on complex phenotypes in vivo; this provides a method to evaluate the effects of a single miRNA target. These new regulatory molecules provide broad prospects for the development of novel therapies. We need to explore the influence of single miRNA-target interaction further and develop new methods to regulate miRNA biology. In addition to noncoding RNA molecules, there are also some compounds such as E17241 and *N*-benzothiazolyl-2-benzenesulfonamide that promote cholesterol efflux by enhancing ABCA1 mRNA and protein expression in cells and have been identified as novel ABCA1 expression upregulators [38, 39].

Some substances, including leonurine, E3317, mangiferin, Shen-Hong-Tong-Luo, and celastrol, prevent AS via the oxidative stress pathway or participate in the inflammatory response to enhance ABCA1 function by upregulating the expression of ABCA1 via the peroxisome proliferator-activated receptor gamma (PPAR-*γ*)/liver X receptor alpha (LXR-*α*)/ABCA1 pathway; this promotes cholesterol efflux and reduces lipid accumulation, thus preventing AS [40–44]. Astragalin prevents AS by promoting cholesterol efflux mediated by ABCA1 and ATP-binding cassette transporter G1 ABCG1, in addition to inhibiting the release of proinflammatory mediators [45]. Furthermore, *Myristica fragrans*, a traditional herbal medicine, reduces lipid accumulation and the levels of tumor necrosis factor-*α*, interleukin-6, and interleukin-1*β* and increases interleukin-10 levels, by promoting ABCA1 expression and cholesterol efflux in THP-1-derived macrophages [46] Some cytokines, such as bifunctional supramolecular nanofibers/hydrogels composed of short peptides, consist of a tetrapeptide (SSSR) from the C-region of insulin-like growth factor (IGF)-1, an anti-inflammatory drug naproxen (Npx), and a powerful self-assembling D-peptide (^D^F^D^F). Hydrogels of Npx-^D^F^D^FGSSSR possessed both anti-inflammatory and IGF-1 imitating properties, and it effectively promoted the expression of ABCA1 and ABCG1 to inhibit the pathological progression of AS by regulating cholesterol efflux and inflammation; this may contribute to the development of nanomedicines for the treatment of AS [47].

Hafiane et al. developed an HDL-mimicking peptide based on the C-terminal domain of apolipoprotein E (apoE), which targets ABCA1 for treatment of diseases. And preclinical optimization studies identified CS6253, an ABCA1 agonist peptide with a high safety index, which mimics the ability of apoA1 to promote the formation of ABCA1-mediated nHDL particles and prevents AS in hyperlipidemic mouse models [48–50]. Therefore, they are potentially effective candidate drugs for the prevention and treatment of AS, which suggests a promising therapeutic strategy against AS.

It has also been confirmed that some drugs or metabolites in the body can prevent AS by enhancing ABCA1-mediated cholesterol efflux, e.g., zafirlukast, rosuvastatin, and butyrate, an intestinal microbial metabolite [51–53]. However, the structure and molecular mechanism of ABCA1-mediated lipid transport and nHDL formation are still unknown, but will be the focus of our forthcoming research. The upregulation of ABCA1 gene expression and the inhibition of ABCA1 deactivation are specific aspects that require more investigation.

## 3. LCAT

LCAT (EC2.3.1.43), first described by Glomset in 1962 [54], is a key enzyme in the formation of plasma CEs. Considering that nHDL particles mature via the esterification of cholesterol, LCAT plays an important role in maintaining HDL-C homeostasis. The human LCAT gene (length = 4.5 kb) is located at 16q22 and contains 6 exons, including a coding sequence of 1.5 kb [55]. The mature LCAT with a molecular weight of approximately 67 kDa contains 416 amino acids and is synthesized mainly in the liver [56]. In humans, approximately 90% of plasma CEs are metabolized by LCAT, and this reaction mainly occurs on the surface of HDLs.

LCAT is an important driving force of RCT, and apoA1, which occurs in nHDL, is a powerful activator of LCAT [57]. The interaction between these molecules plays a key role in the maturation of nHDL; initially, apoA1 activates the LCAT reaction and catalyzes the transfer of *sn*-2 acyl of phosphatidylcholine to cholesterol to form a CE; these CEs migrate to and accumulate in the hydrophobic core of HDL particles, thus promoting the maturation of discoid pre-*β*-HDL into spherical *α*-HDL and further promotes cholesterol efflux from atherosclerotic plaques [56, 58]. Furthermore, plasma LCAT binds HDL as well as apoB-containing lipoproteins and actively esterifies cholesterol via *β*-LCAT [59].

### 3.1. Anti-AS Effects of LCAT

The physiological effects of LCAT on AS have not been fully determined [60, 61], and many studies have long assumed that LCAT has an anti-AS effect because it promotes RCT. There is evidence from animal studies to support the hypothesis that LCAT drives RCT to prevent AS, possibly related to cholesterol ester transfer protein (CETP). Next, we will review the effects of increased or decreased LCAT expression on lipoprotein metabolism and AS in various animal models (Table 1).

A previous study found that overexpression of LCAT in mice significantly increases the level of HDL-C, but AS still developed in mice with LCAT overexpression after following a high-fat diet, which suggests that LCAT overexpression alone could not reverse the effect of an atherogenic high-fat diet [60].

However, when LCAT was overexpressed in mice and rabbits expressing CETP, the HDL levels significantly increased, resulting in significant reductions in atherosclerotic lesions [61, 64, 65]. This suggests that the lack of CETP in mice in the former study may have been responsible for the failure to prevent AS. In humans, this protein transfers HDL CEs to apoB-containing lipoproteins, which are then transported to the liver [66]. LCAT deficiency in hamsters can lead to dyslipidemia and ultimately promote the formation of atherosclerotic lesions [63]. Overexpression of LCAT in nonhuman primates increased and decreased the levels of HDL-C and LDL, respectively, similar to what was observed in transgenic rabbits [67]. Overall, results from various animal models suggest a complex interaction between LCAT and AS, and several factors, such as diet, the presence or absence of LDL receptors, CETP, and SR-B1, have been suggested to explain these differences [56, 68]. Moreover, the results of the rabbit study were significantly different from those of the mouse study, while rabbits and nonhuman primates are more similar to humans in terms of lipoprotein metabolism, thus supporting the theory that improving LCAT function is beneficial for preventing AS.

It was reported that increasing the expression of endogenous LCAT gene may increase the level of HDL and prevent AS [69]. LCAT gene-deficient heterozygotes showed low HDL-C levels and the average carotid intima-media thickness in the heterozygote was significantly higher than that in the family control group (0.623 ± 0.13 vs. 0.591 ± 0.08 mm) [70]; LCAT esterified cholesterol by increasing HDL levels and reducing the ability of apoB particles to induce atherogenesis [71]. The results of the above-mentioned studies suggest that increasing the levels of HDL-C-targeting LCAT can reduce the risk of cardiovascular disease and provide supporting evidence for LCAT as a drug target to improve HDL-C levels. Therefore, developing strategies to enhance LCAT activity may be a useful approach in anti-AS therapy.

### 3.2. Recent Progress in the Anti-AS Applications of LCAT

There have been many attempts to enhance LCAT activity through the development of agonistic antibodies, recombinant human LCAT (rhLCAT), and activators. Various studies have indicated that this therapeutic approach is beneficial to patients with AS. Gunawardane et al. developed an agonistic antibody (27C3), which binds to LCAT in humans and crab-eating rhesus monkeys and significantly enhances its activity; a single administration of 27C3 caused a rapid increase in plasma LCAT activity, and a 35% increase in the HDL-C level was observed 32 days after 27C3 administration [72]. This study demonstrated the feasibility of developing anti-human LCAT antibody therapy with good efficacy and pharmacokinetics in nonhuman primates. ACP-501, a rhLCAT, can increase HDL-C levels and promote cholesterol efflux; in an open-label, single-dose escalation study in humans (phase 1b), a single intravenous infusion of ACP-501 showed an acceptable safety profile and caused a significant increase in the dose ratio of LCAT to HDL-C, providing support for the use of rhLCAT in future clinical trials of patients with CAD [73]. After the completion of this study, Bonaca et al. developed a rhLCAT preparation with a longer half-life, MEDI6012, which is currently being tested in phase II clinical trials in patients with CAD, showing that the use of rhLCAT is safe and well tolerated; furthermore, it resulted in a significant increase in the levels of HDL-C [74]. However, compared with the above-mentioned biotherapeutic agents, small molecular activators are cheaper and easier to use.

It has been found that compound A (3-(5-(ethylthio)-1,3,4-thiadiazol-2-ylthio)pyrazine-2-carbonitrile) covalently binds to Cys31 at the active site of LCAT, increases the levels of CE and HDL-C in the plasma of mice and hamsters, and develops sulfhydryl reactive *β*-lactam as a new type of LCAT activator [75, 76]. Recent studies have found that a new oral small molecule LCAT activator, DS-8190, increased HDL-C and reduced the area of atherosclerotic lesions by directly binding to human LCAT protein [77].

In summary, significant progress has been made in anti-AS research involving the promotion of LCAT activity, with rhLCAT already evaluated in clinical trials. This is expected to achieve a new breakthrough in anti-AS therapy via the correction of the lipoprotein profile. The structure and specific molecular mechanisms require investigation, e.g., how LCAT enhances the function of HDL and how it reduces the area of atherosclerotic lesions. Elucidating the mechanism of apolipoprotein-activating LCAT would also facilitate the development of an LCAT activator, which plays a role in the anti-AS effect by promoting HDL-C maturation.

## 4. SR-B1

SR-B1 is the main receptor for HDL and is mainly expressed in the liver, arterial walls, and macrophages. It binds to HDL with apoA1 as an intermediate bridge and mediates the selective uptake of HDL-CE to regulate the plasma levels of HDL-C; this is the last step in RCT. Moreover, SR-B1 and ABCA1 represent the main cholesterol efflux mechanisms in human macrophages and prevent the formation of macrophage foam cells by mobilizing cholesterol.

The human *SCARB1* gene encoding the SR-B1 protein is located on chromosome 12, with a span of more than 86 kb, and contains 13 exons and 12 introns [78]. Members of the scavenger receptor B family have two TMDs, and the N- and C-termini of the protein are located in the cell [79]. SR-B1, a glycoprotein located on the cell membrane, contains 509 amino acids and has a molecular weight of approximately 57 kDa [80]. The topological structure of SR-B1 is horseshoe-shaped; the N- and C-termini, containing 9 and 44 amino acid residues, respectively, represent the cytoplasmic domain of SR-B1; adjacent to the N- and C-termini are TMDs with 22 and 23 amino acid residues, respectively; outside the cell membrane, connected to the two TMDs, is a large extracellular domain composed of 403 amino acid resides [81].

SR-B1-mediated selective uptake of HDL-CE is a two-step process: first, cholesterol-rich donor lipoprotein particles bind to SR-B1, followed by the transfer of CE from the lipoprotein particles to the plasma membrane, resulting in the biliary excretion of cholesterol [14, 82]. apoA1 acts as a ligand, interacting with SR-B1 during the transfer of HDL-CE, and the lack of apoA1 leads to a decrease in SR-B1-mediated selective CE uptake from HDL particles [14, 83]. Thus, the pivotal role of SR-B1 in human lipoprotein metabolism and AS makes it a novel target for the prevention and/or treatment of atherosclerotic cardiovascular disease.

### 4.1. Anti-AS Effects of SR-B1

SR-B1 is widely expressed in a variety of cell and tissue types throughout the body, but recent studies have shown that overexpression of SR-B1 can inhibit atherosclerotic plaque formation in liver cells and macrophages [84]. Adenovirus overexpression of hepatic SR-B1 reduced plasma HDL-C levels and prevented AS in high-fat diet-fed LDL-receptor knockout mice [85]. Feeding a high-cholesterol diet to hepatic SR-B1 knockout mice resulted in a significant accumulation of HDL particles, dysfunctional HDL metabolism, and accelerated atherogenesis [86]. Deficiency of SR-B1 and apoE in mouse macrophages results in dyslipidemia, accelerated AS, myocardial infarction, and premature death [87]. Recent studies have shown that SR-B1 inhibits the development of atherosclerotic lesions by controlling macrophage apoptosis in a macrophage apoptosis inhibitor-dependent manner [88]. This demonstrates that the protective role of the liver and macrophage HDL receptor, SR-B1, has been established in mouse models. Furthermore, recent genomic analyses have shown that SR-B1 has a protective effect against AS in humans, as carriers of *SCARB1* variants with associated SR-B1 dysfunction were shown to be at an increased risk of cardiovascular disease [89]. Overall, the aforementioned studies demonstrate the protective effect of SR-B1 against AS. The multiple reported functions of SR-B1 suggest its potential as a feasible therapeutic target in anti-AS therapy.

### 4.2. Recent Progress in the Anti-AS Applications of SR-B1

Most researchers believe that SR-B1 gene polymorphisms are significantly associated with plasma total cholesterol levels and AS. As early as 2010, it was shown that the single nucleotide polymorphism (SNP) rs10846744 variant of *SCARB1* was significantly associated with common carotid artery intimal thickening in different racial/ethnic groups, especially in women [90]. By comparing the genotypes of 295 patients with coronary heart disease, 302 patients with cerebral infarction, and 312 healthy controls matched for age and sex, it was concluded that the C allele of rs10846744 and the C allele of rs2278986 may be the risk factors and protective factors for coronary heart disease, respectively [91]. The synonymous mutation of *SCARB1* exon 8 rs5888 significantly reduces SR-B1 protein expression and in vitro function by affecting the secondary structure and protein translation of SR-B1 RNA, thereby increasing the risk of AS [92, 93]. Other studies have shown that allele A of SR-B1 exon 1 in male patients with CAD can lead to the increase of serum HDL-C and apoA1 levels; thus, the SR-B1 exon 1 polymorphism may be related to the susceptibility to CAD and the severity of coronary heart disease in the Tianjin Han population [94]. Impaired SR-B1 function caused by a rare *SCARB1* variant, P376L, increases the risk of AS [89]. Therefore, there is growing evidence that the genetic variations that affect the regulation of SR-B1 function may increase the risk of AS.

A review of the above-mentioned studies reveals that upregulating *SCARB1*, increasing the expression level of its protein, or directly enhancing the activity of SR-B1 is the recent focus area in anti-AS therapy research. Urolithin B and 1,2,3,4,6-penta-*O*-galloyl-*β*-D-glucose (PGG) were found to increase cholesterol efflux from cholesterol-rich macrophages to HDL granules by enhancing the expression of SR-B1 and ABCA1 proteins [95, 96]. The beneficial effects of urolithin B and PGG in animal models motivate the further development of new drugs for the prevention and treatment of AS in humans. Recently, extract of *Pandanus tectorius* fruit, a candidate anti-AS agent from natural resources, demonstrated anti-AS and anti-hypercholesterolemic effects by upregulating the gene expression of SR-B1 and downregulating the levels of 3-hydroxy-3-methylglutaric acid, indicating that it can be used as a preventive agent for hypercholesterolemia and AS [97]. Farnesoid X receptor (FXR) activated by obeticholic acid (OCA) increases the expression of SR-B1 in the liver, and recent studies have shown that the combination of OCA and the LXR agonist, GW3965, can significantly increase the levels of mRNA and SR-B1 in the liver of hamsters with hypercholesterolemia, providing direct evidence that the synergistic activation of SR-B1 gene transcription by FXR and LXR plays a role in hepatic lipid metabolism [98]. Human SR-B1 gene transcription, which may also be uniformly activated by FXR and LXR, is mediated by currently unknown regulatory sequences that will be studied in the future.

In summary, it was found that *SCARB1* gene polymorphisms may contribute to the genetic susceptibility to coronary heart disease, and various study results support the beneficial effects of *SCARB1* on human cardiovascular health. Therefore, elucidation of the molecular links between SR-B1 dysfunction and increased susceptibility to AS in humans, including adaptor proteins, signaling molecules, and transcriptional regulators, would establish SR-B1 as a key target for reducing the risk of atherosclerotic cardiovascular disease.

## 5. Conclusions

AS is a vascular disease driven by cholesterol accumulation and inflammation. The treatment approach of removing cholesterol from the blood circulation via the RCT pathway shows promise for the prevention and treatment of AS. Our understanding of ABCA1, LCAT, SR-B1, and other receptor proteins and transporters in RCT has improved, but their anti-AS effects and mechanisms remain unclear. Therefore, the development of anti-AS drugs targeting these molecules still has a long way to go and is expected to become a major research focus in anti-AS therapy in the future.

## Figures and Tables

**Table 1:** Animal models exploring the role of the LCAT gene in cholesterol metabolism.

Animal	Model	Construction	LCAT gene	LCAT activity	HDL-C concentration	AS	Reference
Mice	Transgenic	—	Overexpressed	↑	↑	↑	[60]
Mice	Transgenic	LCAT-Tg was hybridized with CETP-Tg mice	Overexpressed	↑	↑	↓	[61]
Mice	—	LCAT defective type	Knockout	↓	↓	↓	[62]
Hamster	—	LCAT gene mutation	Loss	Loss	↓	↓	[63]
Rabbits	Transgenic	Genomic hLCAT with its own promoter and 3′-flank	Overexpressed	↑	↑	↓	[64]
Squirrel monkey	Virus infection	hLCAT in adenoviruses	Overexpressed	↑	↑	↓	(65)
